# The Conference Habit

**Published:** 1920-06-19

**Authors:** 


### THE MATRONS' AND SISTERS' DEPARTMENT.

### THE CONFERENCE HABIT.

The occasions on which nurses meet for mutual discussion and expression of opinion have multiplied of late years in response to general demand, and the ability to take part in conferences has grown proportionately. The days when nurses were really inarticulate are over. At the present moment many of the women in the nursing world who have a message to give are exceedingly well qualified to deliver it, and most are capable of expressing their opinion lucidly for or against. It is undoubtedly a very healthy sign of life in a profession when its members seek occasions for discussion. It shows that there is growth as well as freedom for the development of the new shoots. When many are eager to hear, and some are quite ready to expound new methods of working out the old ideals, the spirit of mechanical routine which is most to be dreaded in any profession disappears and the difference is felt from the lowest rung of the ladder to the highest. The professional congress or conference is a marked feature in science, in medicine, in the churches.

The latest comer among the professions is nursing. It is still in the experimental stage, and its principles are being elaborated day by day and year by year through the efforts of those who practise it. Nursing is a growing and continually changing profession. It is the hand-maid of medicine and surgery, yet is distinct from either. It can never reach its highest stage of development unless its exponents are ready continually to demonstrate the best paths to follow, the easiest way to overcome impediments, the wisest methods of securing the end in view. It is this which the nursing conference at its best can effect.

The particular value of the conferences organised through the College of Nursing is that they form by far the best medium for bringing nurses into touch with modern aspects of their profession. Much has been written about nursing, but it remains true that the things most worth knowing about it have never been committed to paper. It began as an oral tradition, and as such it has largely remained to this day. Its teachers impart it by word of mouth and practical demonstration. Those who learn are often unaccustomed to gather instruction from books, and even when the pupil has mastered all that could be written about nursing it is as nothing. Nursing as an art has to be lived rather than learned by rote. Its tenets are never complete, and every change in social conditions, every change in medical and surgical knowledge leads to fresh developments of nursing. Hence there is at all times, and in particular at the present time when so many changes are occurring, a great many nursing matters under discussion which are hidden from nurses in general. There are besides many divergent modes of administration. Much is in the melting-pot. And out of this seething mass of opinion some is merely transient and can be shown to be undesirable, some is of permanent value and cannot too soon be recognised as such. The testing-ground for all these things is the conference. Listening to the papers prepared out of ripe experience, contributing criticism, question, or counter experience, nurses from different parts of the country aid in forming nursing opinion. Good seed germinates in far-distant quarters, while the merely plausible husks get exposed and crumble away.

The uses of the local centres in developing the talent necessary for successful conferences is very conspicuous. Within the last few years women as a whole have grown less afraid of hearing their own voices or stepping on to a platform, and the nursing profession has been found to possess not a few members whose talent for cultivated, good-humoured, and telling oratory is a distinct asset. There is still something to be accomplished in this direction. There are still occasions when well-informed women submit to sit still and listen to pernicious talk because they have never learned the simple art of debate which would enable them to put saner views before the audience. In a country where everything is settled by meetings, councils, and committees women must either learn to speak or run the risk of tacitly conniving at error.

In former days the most able matrons were often the most persistently voiceless, partly in consequence of the Victorian tradition of silence in which the great foundress lived her life, and partly for lack of opportunity. There is little doubt that this excessive reticence retarded progress. Wisdom and experience accessible in no written page were locked away. True, the fruits were handed down through sisters trained to carry on the tradition, but help was not available for the numerous contemporaries who wanted advice and looked for it in vain. The nursing conference, as we have it to-day, supplies the want and wipes away from the leaders of the profession the reproach of living too exclusively each for her own little world.

